# Matter-Aggregating Systems at a Classical vs. Quantum Interface

**DOI:** 10.3390/e27030273

**Published:** 2025-03-06

**Authors:** Adam Gadomski, Natalia Kruszewska

**Affiliations:** Group of Modeling of Physicochemical Processes, Institute of Mathematics and Physics, Faculty of Chemical Technology and Engineering, Bydgoszcz University of Science and Technology, 85-796 Bydgoszcz, Poland; nkruszewska@pbs.edu.pl

This editorial refers to the contents of the eight papers published under a common flagship of matter-aggregating systems at a classical vs. quantum interface. Seven out of eight papers are collected in the Special Issue (SI) (https://www.mdpi.com/journal/entropy/special_issues/matter_aggreg, accessed on 27 February 2025), while the eighth, albeit formally not included, is inevitably related to the topical survey presented in the current SI; thus, it ultimately deserves, without a doubt, its intentional inclusion in the SI.

In the underlying Special Issue (SI), covering a very extensive topical survey of matter-aggregating systems across diverse physical space, time, object-size, and energy scales (see https://www.mdpi.com/journal/entropy/special_issues/matter_aggreg), one may directly jump into the items listed below and presented in Table 1 to summarize the main message addressed by the SI.

The topical range of contributions to this SI on matter-aggregating systems at a classical-quantum realm are broad, and can be figuratively described as polydispersive, in terms of the multitude of the physical scales uncovered, as well as in how they delve, if at all, into the passage between classical and quantum physical descriptions/approaches, meeting at the nanoscale, thus, at the objects’ characteristic dimensions of 10 hydrogen atoms (when “glued” together linearly one by one) up to a hundred of *nm*, ultimately.

The nanoscale per se (*1–100 nm*) has been typified as a nexus between the classically uncovered physical principles of the macroscale matter aggregation—based on non-equilibrium statistical thermodynamics—as having implicitly involved a) Newton’s laws, somehow (seemingly) hidden behind the molecular-dynamics experiments [1], and b) quantum-mechanics’ subtle, albeit unintuitive, reasoning, expressed (for simplicity and brevity) by the quantum-size effect, assigned to low-dimensional (Van der Waals-type) heterostructures [2].

The so-called conundrum of each matter-aggregation physical description appears to be a puzzle, at which point a scale is designated to commence effectively, whether this begins at a mesoscale (typically) or at one of the two adjacent scales, namely either at the macroscale (see item H from column #1 of Table 1) or at its microscale counterpart [3] (see items D, F, G from column #1 of Table 1, all included in the SI).

In Table 1, one can find mesoscopic but toward-quantum-interface-oriented approaches (see items, A–C and E) that unveil dynamics of soft matter and biomatter systems in a granules-involving and orderly manner (items A and B), as well as approaches expressing a biophysical point-of-view on matter aggregation (with a Fokker-Planck and Smoluchowski-type picture behind it), with virtual symmetry-breaking and chaotic behaviors involved (cf. C and E from Table 1, respectively). In particular, one can plunge into a numerical-simulation demonstration of an advanced but practical algorithmic analysis (based on recurrence plots and time series) of seed mucilage data (item E in Table 1) that reveal features of the system’s dynamics, namely, temperature-dependent regions with different dynamics concerning hydrogen bonds, and regions of stable oscillation of increments of several hydrophobic–polar interactions [4]. 

It is also worth noting that a certain concise-in-form forerunner of this SI appeared in the same Journal as a commentary in early 2023, cf. [5], and is referenced therein.

To summarize, this SI, as briefly presented in Table 1, has primarily focused on a very exhaustive topical survey, touching upon principles of matter aggregation, through their physically relevant size, length, time, and energy, as well as interactions scales. The range of topics addressed in the SI (see also ref. [6]) present a very expanded field, from the early universe to complex organisms (cf., review C in Table 1). In addition, the collected material of the SI can provoke us to foresee that a continuation of these studies, due to presumptions of improper statistical-mechanical incoherent mixing and the inclusion of many-body intermolecular (or, interatomic) interactions, ought to take into account principles of non-extensive thermodynamics (cf. an overview [7]).

To recap concisely, matter aggregation and cluster–cluster formation play a crucial role in the transition from classical to quantum stochastic description, ultimately leading to the formation of nanostructures with reduced dimensionality. This process involves several key mechanisms and phenomena, such as chaotic and quantized reaction pathways, Markov state (or, non-Markovian [8]) models, and/or Ising-type model analogies, as studied by the Monte Carlo method [9], with a plausible temperature- and interaction-type-dependent (also, memory-involving) classical vs. quantum excursion toward nonequilibria [10].

## Figures and Tables

**Table 1:** In the table, in column #1, the topics covered by the SI are listed in their order of appearance (cf. List of Contributions). In column #2, the main keyword is assigned to the corresponding topic, whereas in column #3, the basic signature of the classical vs. quantum passage is included.

#1	#2	#3
A/(Nano)Granules-Involving Aggregation	Nanoscale quantum-size effect	Heisenberg uncertainty relation
B/Molecular Dynamics Simulations of C60 Fullerenes	(Non)resonance bonds	Electron delocalization between different atoms within a molecule
C/Symmetry-Breaking Exploring Energy Dissipation across Diverse Aggregation Scales (review)	Emergence of life in complex systems and organisms	Enantiomeric amino acids and proteins, and their aggregates
D/Bosonic Gases under Harmonic Confinement	Canonical vs. grand canonical ensemble	Bose–Einstein condensation
E/Aggregation of Seed Mucilage Components	Recurrence plots uncovering dynamics of biological molecules	Non-covalent interactions and bonding
F/Ising Paradigm in Isobaric Ensembles (review)	Local entropic effects	Compressible spin cells
G/Particles with Fermionic Internal Degrees of Freedom	Mach–Zehnder interferometer and Hubbard dimer	Interference of fermionic-type particles
H/Aggregation in Systems of Self-Propelled Rods	Low-symmetry interaction between rods	Dynamics of self-propelled rods as resembling a vortex involving boson systems behavior
